# Supplementary material for: Distinct patterns of microbial association across deep-sea corals from the Western Pacific Magellan Seamounts
Source: Microbiol Spectr. 2025 Dec 4;14(1):e02093-25. doi: 10.1128/spectrum.02093-25 (PMC12772408; doi:10.1128/spectrum.02093-25)
Supplement: Supplemental figures — Figures S1 to S4. [file spectrum.02093-25-s0001.docx]

**Supplementary Materials**

**Supplementary File 1.** Metadata of the coral samples.

**Supplementary File 2.** The 28S rRNA gene sequences of the coral samples.

**Supplementary File 3.** The Cytochrome c oxidase subunit I (COI) gene sequences of the coral samples.

**Figure S1.** Phylogeny of Hexacorallia corals based on the 28S rRNA gene.

**Figure S2.** Phylogeny of Hexacorallia corals based on the COI gene.

**Figure S3.** Phylogeny of Octocorallia corals based on the 28S rRNA gene.

**Figure S4.** Phylogeny of Octocorallia corals based on the COI gene.

**Figure S1.** Phylogeny of Hexacorallia corals based on 28S rRNA gene. Sequences generated in the current study are highlighted in green and voucher sequences are highlighted in red. The root of the Hexacorallia trees was positioned between the families Cladopathidae and Schizopathidae.

**Figure S2.** Phylogeny of Hexacorallia corals based on COI gene. Sequences generated in the current study are highlighted in green and voucher sequences are highlighted in red. The root of the Hexacorallia trees was positioned between the genera *Parantipathes* and *Bathypathes*.

**Figure S3.** Phylogeny of Octocorallia corals based on 28S rRNA gene. Sequences generated in the current study are highlighted in green and voucher sequences are highlighted in red. The root of the Octocorallia trees was placed between the orders Malacalcyonacea and Scleralcyonacea.

**Figure S4.** Phylogeny of Octocorallia corals based on COI gene. Sequences generated in the current study are highlighted in green and voucher sequences are highlighted in red. The root of the Octocorallia trees was placed between the orders Malacalcyonacea and Scleralcyonacea.
